# Supplementary material for: Long-Term Circulation of Atypical Porcine Pestivirus (APPV) within Switzerland
Source: Viruses. 2019 Jul 17;11(7):653. doi: 10.3390/v11070653 (PMC6669711; doi:10.3390/v11070653)
Supplement: Supplementary file 1 [file viruses-11-00653-s001.zip › Kaufmann-et-al-2019_Figure-S1.pdf]

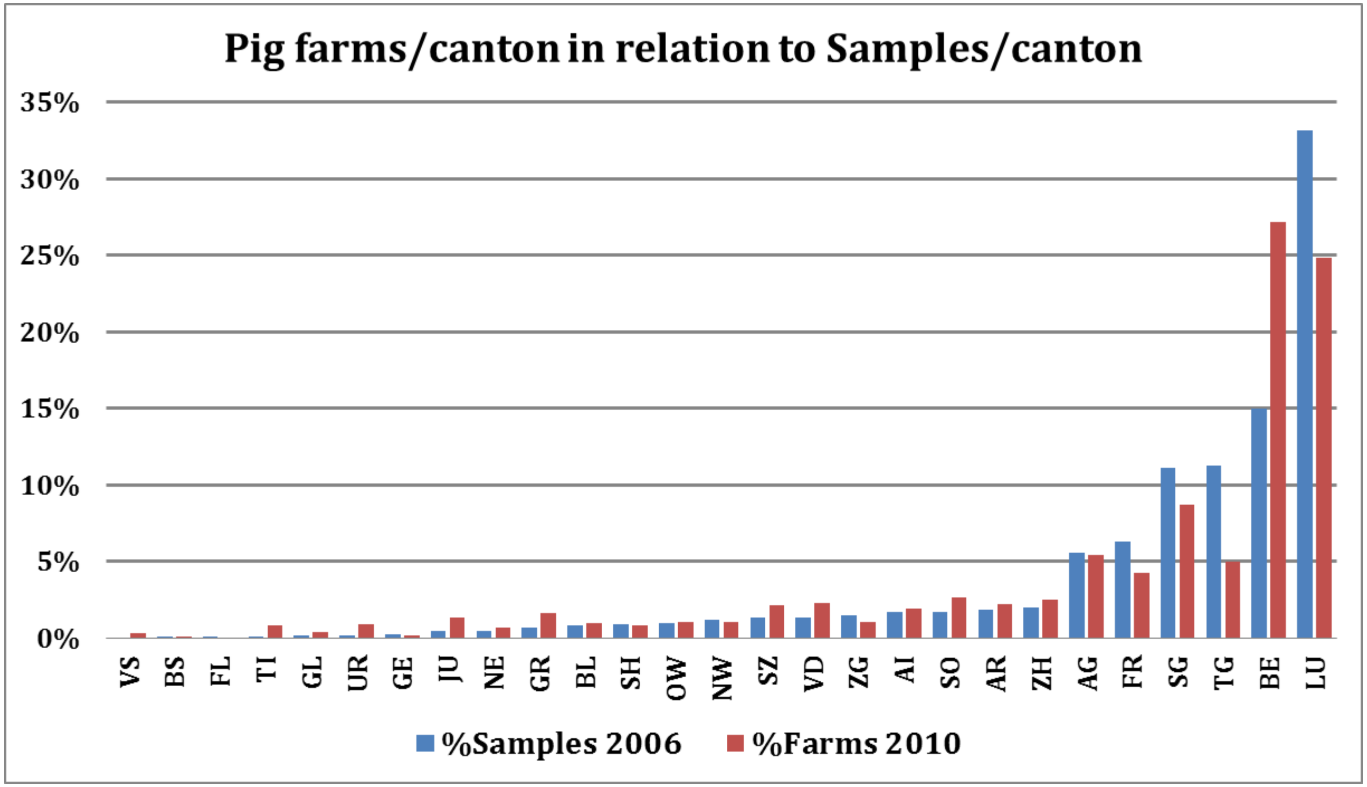

Figure S1. Relative numbers of pig farms per canton (data available for 2010) in relation to the number of samples analyzed in 2006 per canton in Switzerland.
